# 3-[(4-Fluoro­phen­yl)sulfon­yl]-5-iodo-2-methyl-1-benzofuran

**DOI:** 10.1107/S1600536810034069

**Published:** 2010-08-28

**Authors:** Hong Dae Choi, Pil Ja Seo, Byeng Wha Son, Uk Lee

**Affiliations:** a Department of Chemistry, Dongeui University, San 24 Kaya-dong Busanjin-gu, Busan 614-714, Republic of Korea; b Department of Chemistry, Pukyong National University, 599-1 Daeyeon 3-dong, Nam-gu, Busan 608-737, Republic of Korea

## Abstract

In the title compound, C_15_H_10_FIO_3_S, the 4-fluoro­phenyl ring makes a dihedral angle of 72.27 (6)° with the mean plane of the benzofuran fragment [mean deviation of 0.014 (2) Å from the plane defined by the nine constituent atoms]. In the crystal, pairs of weak inter­molecular C—H⋯O hydrogen bonds link the mol­ecules into centrosymmetric dimers, which are further linked *via* an aromatic π–π inter­actions between the iodo­benzene rings of neighbouring mol­ecules [centroid–centroid distance = 3.569 (3) Å].

## Related literature

For the pharmacological activity of benzofuran compounds, see: Aslam *et al.* (2006 ▶); Galal *et al.* (2009 ▶); Khan *et al.* (2005 ▶). For natural products with benzofuran rings, see: Akgul & Anil (2003 ▶); Soekamto *et al.* (2003 ▶). For the structures of related 3-(4-fluoro­phenyl­sulfon­yl)-5-halo-2-methyl-1-benzofuran deriv­atives, see: Choi *et al.* (2010**a* ▶,*b* ▶,c* ▶).
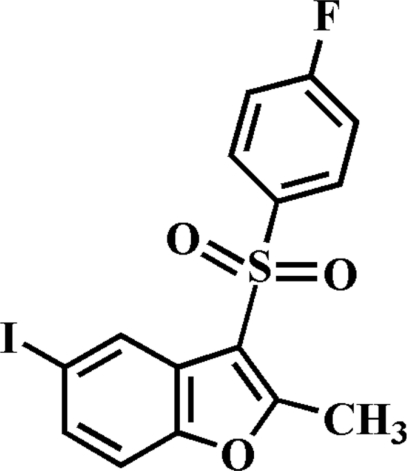


## Experimental

### 

#### Crystal data

C_15_H_10_FIO_3_S
*M* *_r_* = 416.19
Triclinic, 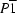

*a* = 7.5324 (3) Å
*b* = 9.4289 (3) Å
*c* = 11.6460 (4) Å
α = 69.687 (1)°
β = 77.639 (2)°
γ = 67.410 (2)°
*V* = 713.06 (4) Å^3^
*Z* = 2
Mo *K*α radiation
μ = 2.41 mm^−1^
*T* = 179 K
0.32 × 0.31 × 0.15 mm

#### Data collection

Bruker SMART APEXII CCD diffractometer
Absorption correction: multi-scan (*SADABS*; Bruker, 2009 ▶) *T*_min_ = 0.516, *T*_max_ = 0.715
12406 measured reflections
3286 independent reflections
3117 reflections with *I* > 2σ(*I*)
*R*_int_ = 0.032

#### Refinement

*R*[*F* ^2^ > 2σ(*F* ^2^)] = 0.026
*wR*(*F* ^2^) = 0.067
*S* = 1.12
3286 reflections
192 parameters
H-atom parameters constrained
Δρ_max_ = 0.46 e Å^−3^
Δρ_min_ = −0.74 e Å^−3^

### 

Data collection: *APEX2* (Bruker, 2009 ▶); cell refinement: *SAINT* (Bruker, 2009 ▶); data reduction: *SAINT*; program(s) used to solve structure: *SHELXS97* (Sheldrick, 2008 ▶); program(s) used to refine structure: *SHELXL97* (Sheldrick, 2008 ▶); molecular graphics: *ORTEP-3* (Farrugia, 1997 ▶) and *DIAMOND* (Brandenburg, 1998 ▶); software used to prepare material for publication: *SHELXL97*.

## Supplementary Material

Crystal structure: contains datablocks global, I. DOI: 10.1107/S1600536810034069/nk2056sup1.cif

Structure factors: contains datablocks I. DOI: 10.1107/S1600536810034069/nk2056Isup2.hkl

Additional supplementary materials:  crystallographic information; 3D view; checkCIF report

## Figures and Tables

**Table 1:** Hydrogen-bond geometry (Å, °)

*D*—H⋯*A*	*D*—H	H⋯*A*	*D*⋯*A*	*D*—H⋯*A*
C11—H11⋯O2^i^	0.95	2.56	3.399 (3)	148

Symmetry code: (i) 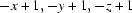.

**Fig. 1.**
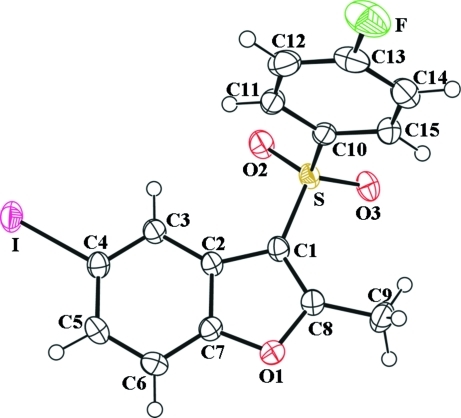
The molecular structure of the title compound with the atom numbering scheme. Displacement ellipsoids are drawn at the 50% probability level. H atoms are presented as a small spheres of arbitrary radius.

**Fig. 2.**
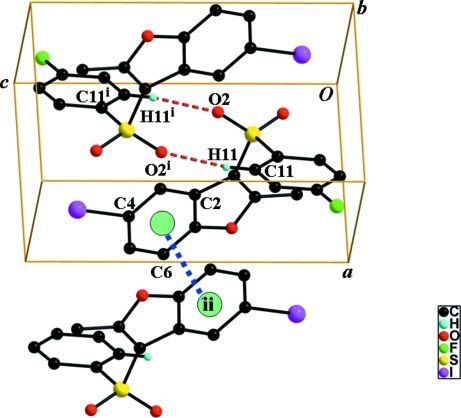
C—H···O and π–π interactions (dotted lines) in the crystal structure of the title compound. Cg denotes the ring centroid. [Symmetry codes: (i) - x + 1, - y + 1, - z + 1; (ii) - x + 2, - y, - z + 1.]

## supplementary crystallographic information

### Comment

Many compounds containing a benzofuran ring system show diverse pharmacological properties such as antifungal, antitumor, antiviral, and antimicrobial activities (Aslam *et al.*, 2006, Galal *et al.*, 2009, Khan *et al.*, 2005). These compounds widely occur in nature (Akgul & Anil, 2003; Soekamto *et al.*, 2003). As a part of our study of the substituent effect on the solid state structures of 3-(4-fluorophenylsulfonyl)-5-halo-2-methyl-1-benzofuran analogues (Choi *et al.*, 2010*a*,*b*,*c*), we report the crystal structure of the title compound (Fig. 1).

The benzofuran unit is essentially planar, with a mean deviation of 0.014 (2) Å from the least-squares plane defined by the nine constituent atoms. The dihedral angle formed by the benzofuran plane and the 4-fluorophenyl ring is 72.27 (6)° . The crystal packing (Fig. 2) is stabilized by weak intermolecular C—H···O hydrogen bonds between the 4–fluorophenyl H atom and the oxygen of the O═S═O unit, with C11—H11···O2^i^ (Table 1). The crystal packing (Fig. 2) is further stabilized by an aromatic π..π interaction between the benzene rings of adjacent molecules, with a Cg···Cg^ii^ distance of 3.569 (3) Å (Cg is the centroid of the C2-C7 benzene ring).

### Experimental

77% 3-chloroperoxybenzoic acid (381 mg, 1.7 mmol) was added in small portions to a stirred solution of 3-(4-fluorophenylsulfanyl)-5-iodo-2-methyl-1-benzofuran (307 mg, 0.8 mmol) in dichloromethane (40 mL) at 273 K. After being stirred at room temperature for 8h, the mixture was washed with saturated sodium bicarbonate solution and the organic layer was separated, dried over magnesium sulfate, filtered and concentrated at reduced pressure. The residue was purified by column chromatography (hexane–ethyl acetate, 1:1 v/v) to afford the title compound as a colorless solid [yield 81%, m.p. 438–439 K; *R*_f_ = 0.71 (hexane–ethyl acetate, 2:1 v/v)]. Single crystals suitable for X-ray diffraction were prepared by slow evaporation of a solution of the title compound in chloroform at room temperature.

### Refinement

All H atoms were positioned geometrically and refined using a riding model, with C—H = 0.93 Å for aryl and 0.97 Å for methyl H atoms. *U*_iso_(H) = 1.2*U*_eq_(C) for aryl and 1.5*U*_eq_(C) for methyl H atoms.

### Crystal data

**Table:**

C_15_H_10_FIO_3_S	*Z* = 2
*M**_r_* = 416.19	*F*(000) = 404
Triclinic, *P*1	*D*_x_ = 1.938 Mg m^−^^3^
Hall symbol: -P 1	Mo *K*α radiation, λ = 0.71073 Å
*a* = 7.5324 (3) Å	Cell parameters from 9807 reflections
*b* = 9.4289 (3) Å	θ = 2.5–27.5°
*c* = 11.6460 (4) Å	µ = 2.41 mm^−^^1^
α = 69.687 (1)°	*T* = 179 K
β = 77.639 (2)°	Block, colourless
γ = 67.410 (2)°	0.32 × 0.31 × 0.15 mm
*V* = 713.06 (4) Å^3^	

### Data collection

**Table:**

Bruker SMART APEXII CCD diffractometer	3286 independent reflections
Radiation source: rotating anode	3117 reflections with *I* > 2σ(*I*)
graphite multilayer	*R*_int_ = 0.032
Detector resolution: 10.0 pixels mm^-1^	θ_max_ = 27.5°, θ_min_ = 1.9°
φ and ω scans	*h* = −9→9
Absorption correction: multi-scan (*SADABS*; Bruker, 2009)	*k* = −12→12
*T*_min_ = 0.516, *T*_max_ = 0.715	*l* = −15→14
12406 measured reflections	

### Refinement

**Table:**

Refinement on *F*^2^	Primary atom site location: structure-invariant direct methods
Least-squares matrix: full	Secondary atom site location: difference Fourier map
*R*[*F*^2^ > 2σ(*F*^2^)] = 0.026	Hydrogen site location: difference Fourier map
*wR*(*F*^2^) = 0.067	H-atom parameters constrained
*S* = 1.12	*w* = 1/[σ^2^(*F*_o_^2^) + (0.0304*P*)^2^ + 0.3949*P*] where *P* = (*F*_o_^2^ + 2*F*_c_^2^)/3
3286 reflections	(Δ/σ)_max_ = 0.001
192 parameters	Δρ_max_ = 0.46 e Å^−^^3^
0 restraints	Δρ_min_ = −0.74 e Å^−^^3^

### Special details

**Table:**

Geometry. All esds (except the esd in the dihedral angle between two l.s. planes) are estimated using the full covariance matrix. The cell esds are taken into account individually in the estimation of esds in distances, angles and torsion angles; correlations between esds in cell parameters are only used when they are defined by crystal symmetry. An approximate (isotropic) treatment of cell esds is used for estimating esds involving l.s. planes.
Refinement. Refinement of F^2^ against ALL reflections. The weighted R-factor wR and goodness of fit S are based on F^2^, conventional R-factors R are based on F, with F set to zero for negative F^2^. The threshold expression of F^2^ > 2sigma(F^2^) is used only for calculating R-factors(gt) etc. and is not relevant to the choice of reflections for refinement. R-factors based on F^2^ are statistically about twice as large as those based on F, and R- factors based on ALL data will be even larger.

### Fractional atomic coordinates and isotropic or equivalent isotropic displacement parameters (Å^2^)

**Table:**

	*x*	*y*	*z*	*U*_iso_*/*U*_eq_	
I	0.74536 (3)	0.07895 (2)	0.834688 (14)	0.03858 (8)	
S	0.46135 (8)	0.39074 (6)	0.29783 (5)	0.02623 (12)	
F	1.0151 (3)	0.7277 (2)	0.0740 (2)	0.0570 (5)	
O1	0.7819 (2)	−0.06398 (19)	0.35078 (15)	0.0287 (3)	
O2	0.3608 (3)	0.4405 (2)	0.40446 (17)	0.0371 (4)	
O3	0.3545 (3)	0.4119 (2)	0.20121 (16)	0.0342 (4)	
C1	0.6012 (3)	0.1889 (3)	0.3496 (2)	0.0253 (4)	
C2	0.6714 (3)	0.1055 (2)	0.4696 (2)	0.0229 (4)	
C3	0.6569 (3)	0.1441 (3)	0.5777 (2)	0.0259 (5)	
H3	0.5818	0.2481	0.5853	0.031*	
C4	0.7570 (3)	0.0235 (3)	0.6732 (2)	0.0261 (5)	
C5	0.8659 (3)	−0.1312 (3)	0.6658 (2)	0.0292 (5)	
H5	0.9309	−0.2100	0.7342	0.035*	
C6	0.8799 (3)	−0.1707 (3)	0.5599 (2)	0.0281 (5)	
H6	0.9530	−0.2755	0.5532	0.034*	
C7	0.7821 (3)	−0.0502 (3)	0.4643 (2)	0.0243 (4)	
C8	0.6729 (3)	0.0833 (3)	0.2820 (2)	0.0273 (5)	
C9	0.6640 (4)	0.0959 (3)	0.1531 (2)	0.0368 (6)	
H9A	0.6471	−0.0008	0.1502	0.055*	
H9B	0.7842	0.1063	0.1045	0.055*	
H9C	0.5547	0.1906	0.1192	0.055*	
C10	0.6315 (3)	0.4907 (2)	0.2315 (2)	0.0253 (4)	
C11	0.7203 (4)	0.5247 (3)	0.3064 (2)	0.0323 (5)	
H11	0.6913	0.4938	0.3931	0.039*	
C12	0.8523 (4)	0.6048 (3)	0.2529 (3)	0.0398 (6)	
H12	0.9165	0.6284	0.3022	0.048*	
C13	0.8877 (4)	0.6489 (3)	0.1269 (3)	0.0376 (6)	
C14	0.7985 (4)	0.6179 (3)	0.0512 (3)	0.0357 (6)	
H14	0.8248	0.6520	−0.0356	0.043*	
C15	0.6704 (4)	0.5361 (3)	0.1045 (2)	0.0288 (5)	
H15	0.6089	0.5110	0.0547	0.035*	

### Atomic displacement parameters (Å^2^)

**Table:**

	*U*^11^	*U*^22^	*U*^33^	*U*^12^	*U*^13^	*U*^23^
I	0.04426 (12)	0.04278 (11)	0.02458 (10)	−0.00531 (8)	−0.00975 (7)	−0.01169 (7)
S	0.0244 (3)	0.0270 (2)	0.0210 (3)	−0.0027 (2)	−0.0043 (2)	−0.0050 (2)
F	0.0535 (11)	0.0454 (9)	0.0756 (14)	−0.0303 (8)	−0.0174 (10)	0.0000 (9)
O1	0.0308 (9)	0.0273 (7)	0.0271 (8)	−0.0070 (7)	−0.0022 (7)	−0.0106 (6)
O2	0.0353 (10)	0.0354 (9)	0.0273 (9)	0.0005 (7)	0.0014 (8)	−0.0097 (7)
O3	0.0306 (9)	0.0384 (9)	0.0295 (9)	−0.0098 (7)	−0.0113 (7)	−0.0022 (7)
C1	0.0234 (11)	0.0277 (10)	0.0209 (10)	−0.0061 (8)	−0.0016 (9)	−0.0057 (8)
C2	0.0193 (10)	0.0251 (9)	0.0202 (10)	−0.0054 (8)	−0.0003 (8)	−0.0051 (8)
C3	0.0241 (11)	0.0268 (10)	0.0222 (11)	−0.0043 (8)	−0.0002 (9)	−0.0075 (8)
C4	0.0241 (11)	0.0313 (10)	0.0212 (10)	−0.0091 (9)	−0.0003 (9)	−0.0069 (8)
C5	0.0260 (12)	0.0283 (10)	0.0260 (11)	−0.0062 (9)	−0.0053 (9)	−0.0011 (9)
C6	0.0257 (11)	0.0231 (9)	0.0313 (12)	−0.0057 (8)	−0.0014 (9)	−0.0065 (9)
C7	0.0231 (11)	0.0270 (10)	0.0225 (10)	−0.0091 (8)	0.0020 (9)	−0.0085 (8)
C8	0.0258 (11)	0.0298 (10)	0.0248 (11)	−0.0083 (9)	−0.0026 (9)	−0.0074 (9)
C9	0.0393 (14)	0.0442 (13)	0.0278 (13)	−0.0091 (11)	−0.0035 (11)	−0.0174 (11)
C10	0.0252 (11)	0.0212 (9)	0.0269 (11)	−0.0014 (8)	−0.0070 (9)	−0.0086 (8)
C11	0.0374 (13)	0.0260 (10)	0.0301 (12)	0.0005 (9)	−0.0125 (10)	−0.0115 (9)
C12	0.0428 (15)	0.0287 (11)	0.0528 (17)	−0.0065 (11)	−0.0202 (13)	−0.0147 (11)
C13	0.0367 (14)	0.0226 (10)	0.0504 (16)	−0.0089 (10)	−0.0097 (12)	−0.0049 (10)
C14	0.0371 (14)	0.0310 (11)	0.0327 (13)	−0.0091 (10)	−0.0047 (11)	−0.0038 (10)
C15	0.0317 (12)	0.0290 (10)	0.0253 (11)	−0.0085 (9)	−0.0064 (9)	−0.0072 (9)

### Geometric parameters (Å, °)

**Table:**

I—C4	2.096 (2)	C6—C7	1.380 (3)
S—O3	1.4362 (19)	C6—H6	0.9500
S—O2	1.4369 (18)	C8—C9	1.478 (3)
S—C1	1.741 (2)	C9—H9A	0.9800
S—C10	1.768 (2)	C9—H9B	0.9800
F—C13	1.352 (3)	C9—H9C	0.9800
O1—C8	1.370 (3)	C10—C11	1.385 (3)
O1—C7	1.373 (3)	C10—C15	1.388 (3)
C1—C8	1.361 (3)	C11—C12	1.390 (4)
C1—C2	1.444 (3)	C11—H11	0.9500
C2—C7	1.398 (3)	C12—C13	1.375 (4)
C2—C3	1.399 (3)	C12—H12	0.9500
C3—C4	1.386 (3)	C13—C14	1.378 (4)
C3—H3	0.9500	C14—C15	1.376 (4)
C4—C5	1.396 (3)	C14—H14	0.9500
C5—C6	1.379 (4)	C15—H15	0.9500
C5—H5	0.9500		
			
O3—S—O2	119.80 (12)	C1—C8—O1	110.4 (2)
O3—S—C1	108.88 (11)	C1—C8—C9	134.2 (2)
O2—S—C1	107.18 (11)	O1—C8—C9	115.4 (2)
O3—S—C10	107.53 (11)	C8—C9—H9A	109.5
O2—S—C10	108.01 (12)	C8—C9—H9B	109.5
C1—S—C10	104.41 (11)	H9A—C9—H9B	109.5
C8—O1—C7	107.05 (17)	C8—C9—H9C	109.5
C8—C1—C2	107.58 (19)	H9A—C9—H9C	109.5
C8—C1—S	126.09 (18)	H9B—C9—H9C	109.5
C2—C1—S	126.25 (18)	C11—C10—C15	121.4 (2)
C7—C2—C3	119.0 (2)	C11—C10—S	119.71 (19)
C7—C2—C1	104.5 (2)	C15—C10—S	118.90 (19)
C3—C2—C1	136.5 (2)	C10—C11—C12	119.1 (2)
C4—C3—C2	116.9 (2)	C10—C11—H11	120.4
C4—C3—H3	121.5	C12—C11—H11	120.4
C2—C3—H3	121.5	C13—C12—C11	118.3 (3)
C3—C4—C5	123.0 (2)	C13—C12—H12	120.8
C3—C4—I	118.01 (17)	C11—C12—H12	120.8
C5—C4—I	119.02 (17)	F—C13—C12	118.8 (3)
C6—C5—C4	120.5 (2)	F—C13—C14	118.0 (3)
C6—C5—H5	119.8	C12—C13—C14	123.2 (3)
C4—C5—H5	119.8	C15—C14—C13	118.3 (2)
C5—C6—C7	116.6 (2)	C15—C14—H14	120.8
C5—C6—H6	121.7	C13—C14—H14	120.8
C7—C6—H6	121.7	C14—C15—C10	119.6 (2)
O1—C7—C6	125.5 (2)	C14—C15—H15	120.2
O1—C7—C2	110.46 (19)	C10—C15—H15	120.2
C6—C7—C2	124.1 (2)		
			
O3—S—C1—C8	28.4 (2)	C1—C2—C7—C6	178.9 (2)
O2—S—C1—C8	159.4 (2)	C2—C1—C8—O1	1.2 (3)
C10—S—C1—C8	−86.2 (2)	S—C1—C8—O1	177.96 (17)
O3—S—C1—C2	−155.4 (2)	C2—C1—C8—C9	−176.1 (3)
O2—S—C1—C2	−24.4 (2)	S—C1—C8—C9	0.7 (4)
C10—S—C1—C2	90.0 (2)	C7—O1—C8—C1	−1.2 (3)
C8—C1—C2—C7	−0.7 (3)	C7—O1—C8—C9	176.7 (2)
S—C1—C2—C7	−177.47 (17)	O3—S—C10—C11	163.83 (18)
C8—C1—C2—C3	177.9 (3)	O2—S—C10—C11	33.2 (2)
S—C1—C2—C3	1.1 (4)	C1—S—C10—C11	−80.6 (2)
C7—C2—C3—C4	0.8 (3)	O3—S—C10—C15	−14.9 (2)
C1—C2—C3—C4	−177.6 (3)	O2—S—C10—C15	−145.54 (18)
C2—C3—C4—C5	−1.1 (4)	C1—S—C10—C15	100.62 (19)
C2—C3—C4—I	178.21 (16)	C15—C10—C11—C12	−0.6 (3)
C3—C4—C5—C6	0.6 (4)	S—C10—C11—C12	−179.36 (18)
I—C4—C5—C6	−178.65 (18)	C10—C11—C12—C13	0.8 (3)
C4—C5—C6—C7	0.2 (3)	C11—C12—C13—F	179.6 (2)
C8—O1—C7—C6	−178.1 (2)	C11—C12—C13—C14	0.1 (4)
C8—O1—C7—C2	0.7 (2)	F—C13—C14—C15	179.3 (2)
C5—C6—C7—O1	178.2 (2)	C12—C13—C14—C15	−1.2 (4)
C5—C6—C7—C2	−0.5 (4)	C13—C14—C15—C10	1.4 (4)
C3—C2—C7—O1	−178.9 (2)	C11—C10—C15—C14	−0.5 (3)
C1—C2—C7—O1	0.0 (2)	S—C10—C15—C14	178.25 (18)
C3—C2—C7—C6	0.0 (3)		

### Hydrogen-bond geometry (Å, °)

**Table:**

*D*—H···*A*	*D*—H	H···*A*	*D*···*A*	*D*—H···*A*
C11—H11···O2^i^	0.95	2.56	3.399 (3)	148.

Symmetry codes: (i) −*x*+1, −*y*+1, −*z*+1.
